# Emerging Techniques for Differentiation of Fresh and Frozen–Thawed Seafoods: Highlighting the Potential of Spectroscopic Techniques

**DOI:** 10.3390/molecules25194472

**Published:** 2020-09-29

**Authors:** Abdo Hassoun, Elena Shumilina, Francesca Di Donato, Martina Foschi, Jesus Simal-Gandara, Alessandra Biancolillo

**Affiliations:** 1 Nofima AS, Norwegian Institute of Food, Fisheries, and Aquaculture Research, Muninbakken 9-13, 9291 Tromsø, Norway; 2 Department of Biotechnology, Norwegian University of Science and Technology (NTNU), 7491 Trondheim, Norway; elena.v.shumilina@gmail.com; 3 Department of Physical and Chemical Sciences, University of L’Aquila, Via Vetoio, Coppito, 67100 L’Aquila, Italy; francesca.didonato3@graduate.univaq.it (F.D.D.); martina.foschi@graduate.univaq.it (M.F.); alessandra.biancolillo@univaq.it (A.B.); 4 Nutrition and Bromatology Group, Department of Analytical and Food Chemistry, Faculty of Science, University of Vigo, Ourense Campus, E-32004 Ourense, Spain; jsimal@uvigo.es

**Keywords:** fluorescence, fish, fraud, spectroscopy, NIR, freezing, thawing, chemometrics, freshness

## Abstract

Fish and other seafood products have a limited shelf life due to favorable conditions for microbial growth and enzymatic alterations. Various preservation and/or processing methods have been developed for shelf-life extension and for maintaining the quality of such highly perishable products. Freezing and frozen storage are among the most commonly applied techniques for this purpose. However, frozen–thawed fish or meat are less preferred by consumers; thus, labeling thawed products as fresh is considered a fraudulent practice. To detect this kind of fraud, several techniques and approaches (e.g., enzymatic, histological) have been commonly employed. While these methods have proven successful, they are not without limitations. In recent years, different emerging methods have been investigated to be used in place of other traditional detection methods of thawed products. In this context, spectroscopic techniques have received considerable attention due to their potential as being rapid and non-destructive analytical tools. This review paper aims to summarize studies that investigated the potential of emerging techniques, particularly those based on spectroscopy in combination with chemometric tools, to detect frozen–thawed muscle foods.

## 1. Introduction

Freshness is an important quality parameter of fish and other seafood products. However, maintaining the freshness and quality of such products is challenging as post-mortem alterations occur rapidly due to high water content and neutral pH values, among other factors [1,2]. A wide range of preservation and/or processing techniques is being developed or has been developed and applied in order to address this challenge and extend the shelf life of fish and fishery products. Among these techniques, freezing has gained widespread popularity during the last two centuries owing to the various advantages offered, such as product safety and sensory quality, as well as the preservation of nutritional value [3]. Moreover, in some cases, freezing of fish and derivative products must be performed in order to ensure conformity with specific regulations. For example, freezing at −20 °C for at least 24 h is compulsory for fish that is consumed raw, such as sushi and sashimi [4]. Recent advances in freezing and frozen storage technologies have improved quality and minimized the damage caused to frozen–thawed products, giving products with similar sensorial characteristics to those that are fresh [5,6,7].

However, fresh fish and fishery products are still preferred by consumers who are willing to pay more for fresh than frozen–thawed products. Therefore, seafood products that had been frozen prior to marketing must be labeled correctly (i.e., previously frozen or “defrosted”); otherwise, it is considered a fraudulent practice [8,9,10,11]. To detect and avoid such fraud, different analytical methods and approaches of analysis have been investigated and developed. These include microscopy and scanning electron microscopy [12,13,14], enzymatic and electrophoretic methods [15,16,17,18,19], and other physicochemical measurements (e.g., protein and lipid oxidation, volatile compounds, water-holding capacity, etc.) [20,21,22,23]. Other methods that have been commonly reported in the literature to detect differences between fresh and frozen–thawed seafood are dielectric properties and impedance technology [24,25,26,27]. Despite their good performance, most of the aforementioned techniques are not suitable for commercial applications due to their significant limitations, such as the destructive nature of measurements, the time required to perform the analyses, and the cost of these analyses.

**Figure 1:**
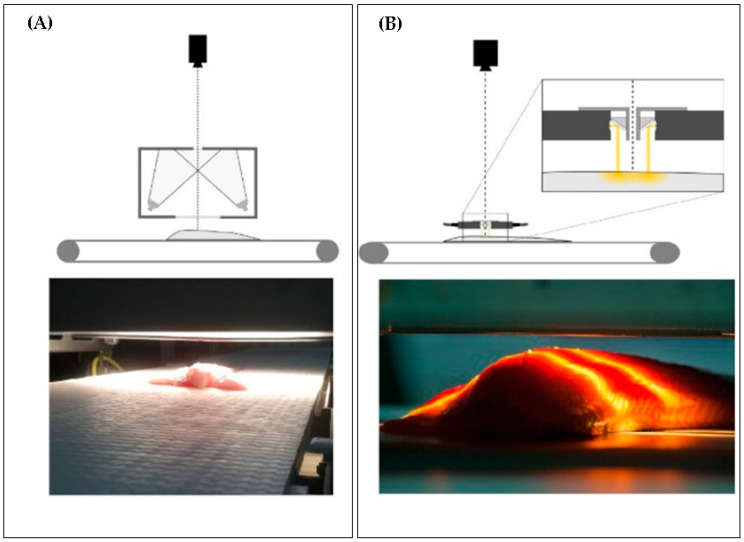
Example of application of spectroscopic measurements (hyperspectral imaging setups) used to scan fish samples on conveyer belts with (**A**) diffuse reflectance mode and (**B**) interactance mode. Reproduced in compliance with CC BY license from Ref. [32].

In the light of the above discussion, it is clear that a need exists for a rapid, non-destructive, and cost-effective method that can be used to ensure the correct labeling of frozen–thawed fish and other seafood. Over the last two decades, several spectroscopic techniques have been successfully applied to determine the freshness of muscle foods rapidly and non-destructively. One of the main advantages of these techniques is the possibility to perform online measurements on conveyor belts, meaning that conversion into industrial applications is possible (Figure 1) [28,29,30,31].

**Figure 2:**
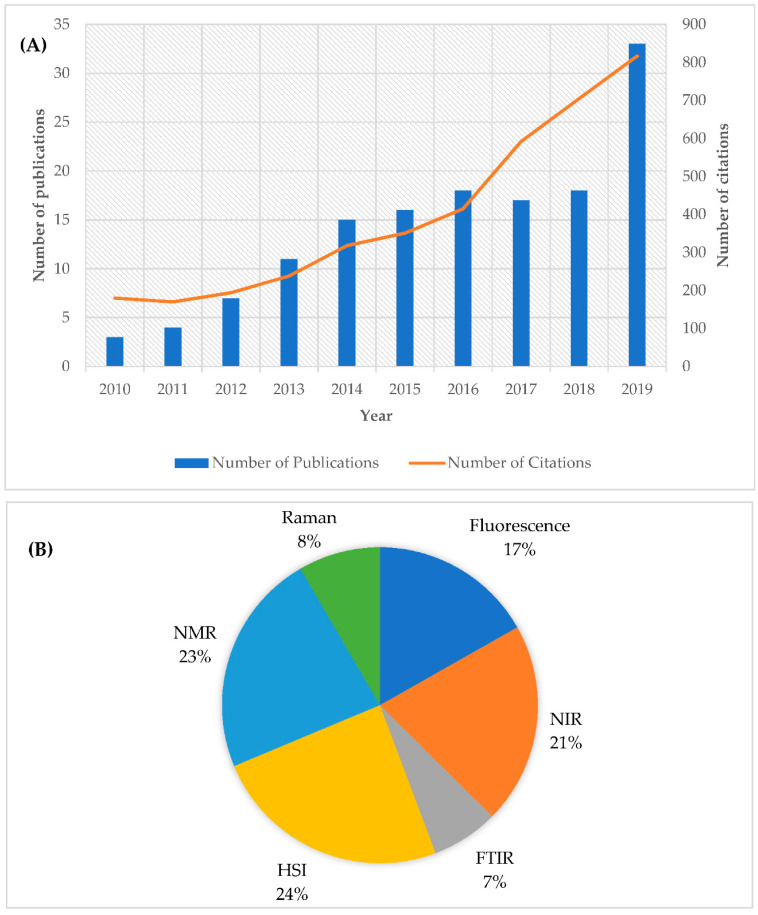
Number of scientific papers and citations (**A**) and publications distributed between the different spectroscopic techniques (**B**) used for detecting freshness or frozen–thawed fish or other seafoods. NMR; nuclear magnetic resonance, NIR; near infrared, HSI; hyperspectral imaging, FTIR; Fourier-transform infrared spectroscopy. Information obtained from the database Scopus (Search criteria: TITLE-ABS-KEY freshness) OR (frozen–thawed) (freezing) AND (fish) OR (seafood) AND (spectroscopy; Figure A or different spectroscopic techniques; Figure B). The data were obtained in August 2020.

That is why spectroscopic-based techniques have gained increasing attention in the last few years. In effect, the number of Scopus-indexed articles, in which spectroscopic techniques have been applied to determine freshness and detect thawed fish or other seafood, as well as the number of citations, have increased significantly during the last decade (Figure 2A). The combined use of visible/near infrared (Vis/NIR), near infrared (NIR), mid-infrared (MIR) [29,33,34,35], Raman spectroscopy [36], hyperspectral imaging (HSI) and multispectral imaging (MSI) [28,37,38], fluorescence [39,40,41], nuclear magnetic resonance spectroscopy [42], and impedance spectroscopy [23] with chemometric tools appears to be an efficient solution for the discrimination between fresh and frozen–thawed fish and fishery products. Our literature review showed that HSI and NMR are the most used techniques, followed by NIR and fluorescence spectroscopy.

Despite the huge number of studies dealing with the topic of discrimination between fresh and frozen–thawed fish and other seafoods by spectroscopic techniques, until now, there have been no reviews that comprehensively summarize these studies. The scientific literature shows that this topic was only addressed briefly as a part of authenticity issues in some previous review papers [8,9,21,43,44,45]. Thus, to the best of our knowledge, this review paper is the first to be totally and exclusively devoted to discussing all the spectroscopic studies that have been published so far on this subject.

## 2. Quality Change Occurring during Freezing, Frozen Storage, and Thawing of Seafoods

As mentioned before, food freezing is an effective and efficient method that has been widely used for food preservation. However, freezing, frozen storage, and thawing could cause biochemical and physical changes that might affect the sensory profile and technological characteristics of fish and other seafood. The rate of such changes depends on several factors including genetics, muscle type, husbandry (diet, handling, stress), and post-mortem treatments (freezing rate, storage temperature and pressure, storage time, thawing method) [18,46]. Various quality indicators can describe compositional and morphological changes that occur during freezing, frozen storage, and thawing. This makes it possible to use different analytical techniques to differentiate between fresh and frozen–thawed products [18,47,48].

In this chapter, we will review the processes that occur during pre-freezing storage and upon muscles freezing, frozen storage, and thawing and their impact on the quality of fish and other seafood.

Biochemical processes occurring in muscles before freezing determine, to a greater extent, their quality after a freezing–thawing cycle. In the pre-rigor state, the cell fluid is tightly bound to the intracellular proteins that limits water diffusion from inside to outside the cell. Post-mortem metabolism will result in a decline in glycogen and ATP levels and an increase in Ca^2+^ concentrations and osmotic pressure in muscle, lowering the muscle’s pH. The onset of *rigor mortis* will cause muscle contraction and the diffusion of some amount of cellular fluids into the extracellular spacing [49]. The resolution of *rigor mortis* is correlated with autolytic changes and the activation of muscle-digesting enzymes. Their activity will define muscle softening. Therefore, the distribution and amount of fluid in intra- and extracellular space, the textural properties of the muscle, its pH, and the activity of digesting enzymes are related to the progression of post-mortem changes and will also define the final quality of frozen–thawed fish and fishery products. The effect of freshness on the fish quality after thawing was discussed recently by Nakazawa and Okazaki [50]. The review discusses that fish frozen before *rigor mortis* has excellent color and texture after thawing. These phenomena are related to the high pH and ATP level in fresh pre-rigor fish meat. The progression of post-mortem processes induces the growth of larger ice crystals in the extracellular space that change the structure of frozen fish muscles and increase the amount of drip loss after thawing.

Freezing causes the formation of sharp-edged ice crystals within the muscle tissue that distorts its structure. Therefore, the measurement and control of ice crystals formation is required to ensure high-quality foods [51]. The rate of freezing and the progression of post-mortem changes will determine ice crystals’ size and their distribution (intracellular and/or extracellular) [48,50]. It has been generally agreed that slow freezing rates cause the formation of large extracellular ice crystals, resulting in significant damage to muscle proteins and cell membranes, whereas fast freezing yields numerous fine ice crystals uniformly distributed mostly at intracellular levels within the muscle [48,50,52]. However, it was reported that ultra-rapid freezing of northern snakehead fillets with liquid nitrogen caused tissue cracking and decreased the water-holding capacity [53]. Therefore, the definition of “fast” and “slow” freezing should be carefully considered together with differences in freezing methods to evaluate how the freezing rate affects the quality of fish. Several other processes during the freezing stage might also affect muscles quality, for instance, the release of digestive enzymes and following protein degradation due to the damage of cell membranes and organelles and freeze-induced concentration of electrolytes, enzymes and pro-oxidants in the unfrozen phase [48,50,52]. In addition to freezing, frozen storage is another critical parameter that can significantly affect the quality of frozen foods. Storage temperature is an important factor that determines the amount of unfrozen water that remains available for chemical reactions, affects protein denaturation, oxidation, and hydrolysis of lipids, and alters the color and odor of fish muscles [50]. The rate of quality changes upon frozen storage varies between species. Tolstorebrov and others discussed the processes occurring in fish upon low and ultra-low storage temperature (lipid oxidation, protein, and metabolites changes) and concluded that the recommended storage temperature for high-quality long-term storage of fish is −35 °C, and a further decrease of storage temperature is unnecessary for industrial needs, although the optimum temperature for frozen storage of meat has been reported to be −40 °C [52,54].

Thawing refers to the melting process of ice crystals. This is a long process compared to freezing and can cause physical and chemical changes in frozen foods, affecting significantly the quality of the product [6]. Empty spaces created after the ice crystals have melted together with protein degradation will affect drip loss, resulting in softer texture, gaps, and changes in taste and flavor [48,50]. The quality changes during thawing are determined by a combination of factors: freezing rate, temperature of frozen storage, and thawing time and temperature [48]. The changes caused by freezing–thawing cycles will affect the post-thawing quality in stored fish and fishery products. Even if the differences in some quality parameters (volatile compounds, biogenic amines, pH, metabolic profiles, or others) will not be significant immediately after thawing, they will become so during post-thawing storage [42,46]. The following section will briefly discuss some of these changes that undergo muscle proteins and lipids during the freezing–thawing cycle.

Protein denaturation and protein oxidation are among the most important changes that occur in frozen foods. Protein oxidation is one of the factors that lead to decreased muscles quality due to reduced tenderness and juiciness, flavor deterioration, and discoloration [55,56]. Protein denaturation during frozen–thawed cycles has been reported by several authors to significantly contribute to the negative textural changes of muscle [49,50,52]. Oxidation can cause protein fragmentation, the formation of disulfide bonds, and protein polymerization; therefore, it will affect the protein solubility, gel-forming ability, surface hydrophobicity, water-binding activity, and reactivity [56]. Protein unfolding and oxidation will alter muscle texture and water-holding capacity. Changes in the texture of seafoods can be described using various indicators: resistance to chewing, hardness, fibrousness, juiciness, and softness/tenderness [48,57].

The water-holding capacity and the related sensory juiciness of fish and other seafoods are also directly associated to the proteins structure and folding [58]. Generally, freezing, frozen storage, and thawing all contribute to a decrease in the water-holding capacity of food muscles [48,49,50]. The moisture content could be evaluated in several ways, including the following quality indicators: drip loss, thaw loss, cooking loss, water-binding capacity, and total moisture content.

Lipid oxidation is one of the main processes that are responsible for the quality deterioration of fish and other seafoods. Lipid oxidation affects color, texture, nutritional value, taste, and aroma, leading to rancidity, which is responsible for off-flavors and unacceptable taste [59]. Fish oil is more susceptible to oxidation compared to mammalian lipids due to the higher percentage of polyunsaturated fatty acids [52]. Lipid oxidation in frozen products leads to the breakdown of lipid and to the formation of a wide array of oxidation products. The nature and proportion of these products can vary widely between foods and depend on the composition of the food as well as the freezing and thawing parameters [59,60]. Lipid oxidation (peroxidation) during frozen storage will be accelerated during thawing [61,62]. The rate of lipid oxidation could be estimated using the thiobarbituric acid reactive substances (TBARS) method. It has been reported in several studies that freezing and thawing increase the TBARS value, and repeated freezing–thawing cycles result in accelerated TBARS accumulation [46,62].

Therefore, the main quality changes occurring in fish and other seafoods during a freezing–thawing cycle are related to changes in the amount and distribution of moisture within the muscle tissue, the release of intracellular components into extracellular space, changes in the muscles’ metabolic composition, the degradation of textural characteristics due to the physical damages or autolytic digestion, protein denaturation, and the oxidation of proteins and lipids.

## 3. Analytical Methods Used to Detect Frozen–Thawed Seafoods

After thawing fish or other seafood products, it is difficult even for experts to differentiate these products from fresh ones, especially when freezing, frozen storage, and thawing are performed correctly [21,63,64]. There are various methods in the available literature that have been applied with variable success to detect thawed muscle foods after freezing and frozen storage. Enzymatic and histological methods are among the most commonly used techniques.

### 3.1. Enzymatic and Electrophoresis Methods

Enzymatic methods of differentiation of frozen–thawed from fresh products are mainly based on the change in enzymatic activity of specific enzymes due to cellular disruption and the release of bound enzymes into the cellular fluid during the freezing and frozen storage. Several enzymes related to mitochondria, red blood cells, and lysosomes have been broadly used as freezing indicators. For example, lactate dehydrogenase was proposed as a marker to distinguish between fresh and frozen–thawed sea bream fillets (*Sparus aurata*) [16]. In another study, lysosomal enzymes were found to be suitable to distinguish between several fresh and frozen–thawed pelagic blue fish species [65]. An earlier study compared the performance of several enzymatic assays, among other approaches, which were performed on the whole fish and skinless fillets for the differentiation between fresh and frozen–thawed plaice (*Pleuronectes platessa*), whiting (*Merlangus merlangus*), and mackerel (*Scomber scombrus*) [18]. The authors recommended the use *a*-D-glucosidase to discriminate between fresh and frozen–thawed fillets. Ethuin and co-authors developed a method to differentiate fresh from frozen–thawed sea bass (*Dicentrarchus labrax*) based on the analysis of the protein composition of the sea bass fillet exudates [15]. In this study, the electrophoresis profiles of fresh versus frozen–thawed fillets were compared; then, spots of interest were selected and characterized by tandem mass spectrometry, which is also known as MS/MS. In another study, nucleotides and related compounds concentration and free calcium concentration, measured in exudates of sea bass fillets, showed the potential of being used as rapid and inexpensive tools to differentiate fresh from frozen–thawed fish [17].

Although enzymatic methods are considered as reliable and precise techniques compared to many other techniques [66], their results are not always consistent and highly depend on fish species and storage conditions [9]. In addition, these methods are only laboratory-applicable, destructive, and they are quite expensive and time-consuming.

### 3.2. Histological Measurements: Changes in Muscle Structure and Microstructure

Histological differentiation between fresh and frozen–thawed fish and fishery products has been reported in several research studies [12,13,14]. These techniques are based upon the investigation of changes in the structure and microstructure of seafoods, occurring during freezing and frozen storage, which is followed by using these changes to distinguish between fresh and frozen–thawed products. As discussed earlier, structural changes and damage in muscle tissues are more obvious with slow freezing compared to rapid freezing. Indeed, slow freezing could cause major structural damages to muscle fibers due to the pressure of large ice crystals between muscle fibres, resulting in the shrinkage of fibers and rupture of endomysium, protein denaturation, and the formation of large gaps between muscle fibers [3,67]. An early study showed an increase in extracellular space and a shrinkage in muscle fibers in frozen–thawed salmon, and the shrinkage and space between fibers increased further during smoking of these previously frozen–thawed fish samples compared to smoked fish prepared from fresh salmon [68].

A histology-based approach for the detection of frozen–thawed products was successfully developed on three of the most common fish species, i.e., gilthead (*Sparus auratus*), red mullet (*Mullus barbatus*), and swordfish (*Xiphias gladius*), and the approach was subsequently validated on samples from 35 fish species, achieving high levels of accuracy, precision, and reproducibility [11]. Later, similar approaches were applied to discriminate between fresh and frozen–thawed products of marinated anchovy (*Engraulis encrasicolus*) fillets [69] and smoked salmon [13]. Recently, four histological parameters were proposed in order to discriminate fresh from frozen–thawed European hake (*Merluccius merluccius*) [12].

### 3.3. Other Detection Approaches

Different other methods may be applied to distinguish between fresh and frozen–thawed fish and other seafoods. DNA degradation in salmon following freezing and thawed was reported to be able to distinguish frozen–thawed fillets [70]. Measurements of volatile composition by solid phase micro-extraction/gas chromatography/mass spectrometry analysis (SPME/GC/MS) was also suggested as markers to differentiate fresh from frozen–thawed products [63]. Microbiological parameters, including total viable counts, counts of Enterobacteriaceae, and psychrotrophic bacteria were measured, among other analyses, in order to distinguish fresh, frozen, and double frozen rainbow trout (*Oncorhynchus mykiss*); the double frozen fish exhibited the lowest microbiological quality [71].

## 4. Detection of Frozen–Thawed Seafoods by Spectroscopic Techniques

Most of the above-discussed methods used for the detection of the frozen–thawed products share several limitations: they are destructive, time-consuming, and can be only used to detect specific parameters or attributes, as they are targeted methods. On the other hand, spectroscopic techniques can be used as non-targeted methods to provide information about multiple attributes of quality rapidly and non-destructively. An overview on the emerging spectroscopic trends for detecting frozen–thawed foods is provided in the sub-paragraphs below. A brief introduction to the specific technique is given in each related section; in Table 1, the spectral intervals investigated by the different techniques are summarized.

### 4.1. UV-Vis and Fluorescence Spectroscopy

UV-Vis and fluorescence spectroscopies [72] are widely recognized as powerful tools in the assessment of food quality due to their sensitivity, specificity, low cost, and rapidity. These techniques belong to spectroscopic methods based on the absorption/emission of a specific radiation. When a molecule in its ground state absorbs a photon in the UV-Vis range (Table 1), it is excited to an electronic level at higher energy, and a typical absorption spectrum can be collected. Fluorescence is complementary to UV-Vis absorption; it occurs when a molecule (excited by the UV-Vis light) dissipates part of its energy (as heat) during vibrational relaxation, and the remaining fraction of the excess energy is lost in the emission of a photon of a lower energy than that of the absorbed photon. Food products include intrinsic fluorophores, such as aromatic amino acids (phenylalanine, tyrosine, and tryptophan), vitamins (vitamin A or retinol, vitamin B2 or riboflavin, vitamin B6 or pyridoxin, and vitamin E or R-tocopherol), and cofactors (nicotinamide adenine dinucleotide; NADH), nucleic acids (ATP), porphyrins (hematoporphyrin), and flavonoids; consequently, fluorescence spectroscopy (in non-destructive mode) represents a strategic tool for sensor technology [43,73]. Multivariate approaches are usually used to handle UV-Vis spectra and complex fluorescence signals. In the latter case, data can be organized into two different structures: an excitation/emission matrix, where rows are the sample mode and columns are given by combinations of excitation–emission wavelengths, or in three-way arrays, called excitation–emission matrix (EEM), where samples are in the first mode, and excitation and emission absorbances are in the other two.

In the literature, it is possible to find different studies where fluorescence is used to discriminate frozen–thawed from fresh muscle foods. For instance, Karoui et al. have used front-face fluorescence spectroscopy (FFFS) to discern fresh-refrigerated from frozen–thawed sea bass fillets [39]. Compared to traditional acquisition of fluorescence spectra (right-angle), FFFS allows the collection of signals from the surface of the sample, thus overcoming limitations related to the right-angle acquisition. This study pointed out the importance of investigating the impact of two different storing conditions on freshness: fillets were firstly frozen–thawed (3 months at −18 °C) and then refrigerated (up to 9 days) and *vice versa*. Principal component analysis (PCA), canonical correlation analysis (CCA), and factorial discriminant analysis (FDA) were applied to the fluorescence data obtained at four excitation/emission wavelengths. In the next step, a multi-block analysis method, namely common components and specific weights analysis (CCSWA), was used to handle the fluorescence data in combination with traditional measurements of color, textural, pH value, and moisture content. This latter approach led to the most accurate results, achieving a total correct classification rate (in external validation) of 94.87%. However, unlike EEM, the use of only one excitation/emission wavelength may not be considered as an efficient procedure to extract all information that could be contained in fish samples.

In the context of food storage analysis, both UV-Vis and fluorescence are widely used to assess the freshness of fish products. For instance, EEM fluorescence spectroscopy has been applied to predict the freshness index of a *k*-value of whole and fillets of horse mackerel (at different initial freshness conditions) directly in the frozen state using partial least square (PLS) regression [74]. This index is calculated as the ratio between the concentration of non-phosphorylated ATP metabolites (HxR, Hx) and the total concentration of all the ATP breakdown/degradation products (ADP, AMP, IMP, HxR, Hx), and it is traditionally obtained by conventional nucleotide-based methods (which are destructive and expensive) assessed in a post-mortem early stage [21,74]. The multivariate calibration models, developed to predict k-indices by PLS, provided a R^2^_CV_ of 0.85 and a root-mean-square error of cross-validation; RMSECV of 11.15% on the whole fish and a R^2^_CV_ of 0.94 and an RMSECV of 7.34% on the fish fillet. Another example is a work conducted by Rahman and others, who developed a high performance (R^2^_pred_ of 0.96 and RMSEP of 5.12%) prediction model for *k*-value determination applying full spectrum partial-least squares (FS-PLS) and interval partial-least squares (iPLS) regression methods on different pre-processed (multiplicative scatter correction, Savitzky–Golay first and second-order derivative) UV-Vis spectra of Japanese dace fish eye fluid [75]. Fluorescence spectroscopy has been also intensively applied as a fundamental tool for the study of protein denaturation, lipid and protein oxidation, and ATP content variation related to refrigeration and freezing (Table 2). However, most of the previous studies have been conducted using standard point-based measurements, while little or no study has been carried out investigating fluorescence imaging that provides information at each single pixel of the scanned sample.

### 4.2. Infrared Spectroscopy and Hyperspectral Imaging

In analytical chemistry, infrared spectroscopy has always been widely used, but, in recent years, it has been in the bullseye. In particular, near-infrared (NIR) spectroscopy and hyperspectral imaging (HSI) have widely spread, thanks to their numerous benefits enhanced by their fruitful coupling with chemometric tools. Briefly, infrared spectroscopy deals with the bending absorption that takes place in the spectral range between 13,000 and 10 cm^−1^. In this domain, it is possible to observe the vibrational modes (hence the name of *vibrational spectroscopy*) such as *stretching* and *bending*. The infrared spectral range is sub-divided into three different regions, near infrared (NIR), mid-infrared (MIR), and far infrared (FIR), whose spectral ranges are reported in Table 1. Historically, the wider investigated range is the MIR; nevertheless, in the last few years, NIR spectroscopy has been “rediscovered” thanks to the development of data analysis tools. In fact, NIR spectra present large bands and are non-selective; these aspects made their exploitation more difficult and/or less effective. Notwithstanding, the application of chemometric methods has allowed maximizing the extraction of information from these signals, and nowadays, NIR is one of the most widely used techniques, in particular in food quality analysis [91]. A Fourier transform (FT) is also necessary to extract information from such spectra. Compared to NIR spectroscopy, Fourier-transform infrared spectroscopy (FTIR) is less used with regard to the detection of frozen–thawed seafoods (Figure 2).

NIR spectroscopy is widely applied for the authentication of the freshness of fish meat (Table 2). For instance, Alamprese and co-workers have used NIR spectroscopy to discriminate fresh and thawed Atlantic mullet filets [34]. In this work, NIR spectra were collected on frozen–thawed and fresh filet samples, and two different classifiers, namely Linear Discriminant Analysis (LDA) and Soft Independent Modeling of Class Analogy (SIMCA), were used to distinguish the investigated objects. The most accurate results were obtained by LDA, which provided correct classification rates (on an external validation set of measurements) of 100% and 97.2% for thawed and fresh samples, respectively. A somewhat similar study, based on NIR spectroscopy, was conducted by Wang and collaborators on tilapia filets [33]. In this study, the authors investigated the possibility of detecting whether fish underwent different cycles of freezing–thawing. In order to achieve this goal, tilapia fillets were frozen and thawed seven times, and NIR spectra were collected at each cycle. NIR signals were randomly divided into a calibration (90 spectra) and a validation set (30 spectra), and PCA and Mahalanobis distance discrimination analysis were used to classify the test set, providing satisfying results. In fact, the (externally validated) accuracy was ≈86% for once frozen–thawed fillets, and ≈93% for samples subjected to diverse freezing cycles. NIR spectroscopy has been used for the authentication of freshness also in cephalopods, as investigated, for instance, by Sannia and others who inspected the coupling of this technique with three different partial least square discriminant analysis (PLS-DA)-based strategies for studying differences between fresh and thawed cuttlefishes over a time period of thirteen days [92]. The study demonstrated that the proposed strategy (NIR spectroscopy coupled with PLS-DA) is suitable to discriminate between fresh and defrosted cuttlefish. In particular, the best results were achieved exploiting the complete spectra (900–1650 nm), leading to a (six-fold cross-validated) correct classification rate of 0.91. On the other hand, this approach led to less satisfactory performances when it came to discerning samples according to the storage time.

In addition to traditional infrared spectroscopy, also, HSI operating in this spectral range is a promising approach that allows obtaining excellent results in this field [5]. This technique provides spectra (in different ranges, depending on the *hyperspectral camera*) for each *pixel* of the image collected on the investigated samples [93]. Despite the availability of cameras that collect spectra in different wavelength domains, the Vis/NIR hyperspectral camera is one of the most widely used. For instance, Shan and collaborators exploited this technique to analyze fresh and frozen–thawed carps [94]. In this study, forty crucian carps were investigated. Of these, six were fresh, eighteen were stored at 4 °C for different days (1, 2, or 3), and sixteen were frozen (−20 °C) for one, two, or three weeks prior to the hyperspectral analysis. The hyperspectral images of the samples were collected on the entire fishes, or on both sides of the filets. Regions-of-interest (ROIs) were extracted and pre-processed by normalization, multiple scattering correction, or derivatives. PLS-DA was used to classify samples into fresh or frozen–thawed; results were remarkable, in particular those on the entire samples, achieving 100% correct classification rates in internal cross-validation. Additionally, the authors used the PLS model to predict the storage time; nevertheless, this approach yielded acceptable but not extremely successfully results, since R_cv_^2^ values of 0.80 for fresh samples and of 0.84 for frozen–thawed fishes were obtained. In a similar study, Zhu and co-authors proposed an HSI-based strategy to discriminate fresh and frozen–thawed halibut [95]. In this study, a PCA model was calculated on the ROIs, and then gray-level co-occurrence matrix analysis was used to select the most relevant texture features. Least squares-support vector machine classification (LS-SVM) was exploited to achieve the purpose of the study. Calculations were made on the extracted spectra, on the selected texture features, and on both. The last approach provided the most satisfying outcome (giving ≈97% of total correct classification rate on the validation set).

One of the main advantages of HSI is the possibility of performing measurements inline/online in real time, making it ideal for industrial applications. Several studies have exploited this concept by running experiments on conveyor belts to mimic industrial environments. For example, one of these studies was conducted on farmed Atlantic salmon fillets placed on a conveyer belt, traveling at the industrial production speed (40 cm/s, corresponding to approximately one fillet per second) using an interactance HSI system, operating in the Vis-NIR region [29]. The results showed that frozen–thawed fillets can be completely separated (100% of correct classification rate in leave one out cross-validation) from fresh ones using the k-nearest neighbor (kNN) classifier applied to pre-processed data with second derivative and a selection of few optimal wavelengths (606 and 636 nm). The discrimination ability of this technique was attributed to the variations in the visible region of the spectrum induced by the oxidation of hemoglobin and myoglobin during the freezing/thawing process. Moreover, light scattering properties, resulted from the heterogeneous structure of fish muscle, were also thought to contribute to the spectral variations observed across the fillet. These results were confirmed later in an extended investigation, in which the same technique was investigated to differentiate between fresh and frozen–thawed cod fillets, as well as classifying the fillets according to different freezing and thawing protocols [28]. Again, classification was made by kNN, and validation was carried out by leave one out cross-validation. The highest accuracy was achieved modeling the full spectra, and it led to a correct classification rate of 100% for fresh samples, and of 98% and 93% for once-thawed and twice-thawed samples, respectively.

It should be added that the fusion of results obtained from different spectroscopic techniques could improve the performance of the built models. For example, NIR penetrates deeper into the sample but has less selectivity compared to MIR. Thus, combining the results from NIR and MIR could be an interesting option in order to combine the merits of the two techniques. The fusion strategy has been tested on several food matrices [96,97,98], while little research can be found in the literature regarding fish and other seafood.

### 4.3. Raman Spectroscopy

Raman spectroscopy is a vibrational technique that, in combination with chemometrics, is suitable and frequently used for food quality control and authentication. For this purpose, analogously to infrared spectroscopy, Raman spectroscopy shows several advantages, since it is a rapid and non-destructive technique that requires a small amount of sample and minimal pre-treatment. Raman spectroscopy consists of collecting the radiations inelastically scattered (Stokes and anti-Stokes scatter) by the samples that contain information about fundamental molecular vibrations and combination bands. To obtain a Raman spectrum, the sample is irradiated by a laser light typically falling in the visible or near infrared region; then, the resulting scattered radiation is collected and analyzed in the spectrometer [99]. The elastic scatter, known as the Rayleigh effect, represents the major and intrinsic component of the scattered light, whereas the Raman effect remains normally a weak phenomenon. Therefore, filters might be used to isolate the Stokes lines from the anti-Stokes and the Rayleigh scattering. A proper selection of the laser source wavelength could reduce the trouble related to fluorescence or enhance sensitivity to the vibrational modes of a certain chromophore group obtaining a resonance Raman scattering [100]. In addition to instrumentation and experimental settings, which influence the Raman spectra and the signal-to-noise ratio, signal pre-processing and chemometric elaboration represent a powerful tool to better exploit the spectral information and to deal with common spectral interferences (such as fluorescence or other background luminescence) that could hide the Raman signal.

Velioğlu and others have evaluated the usefulness of Raman spectroscopy in differentiating fresh and frozen–thawed fish samples of six different species [36]. Fat extraction was performed on fresh and on thawed samples resulting from each of the two frozen–thawed cycles, and Raman spectra, in the range of 200–2000 cm^−1^, were collected using a 785 nm laser source. Smoothing and mean-centering were performed before the application of PCA. Even without building classification models, by means of PCA, it was possible to distinguish samples according to freshness and to attribute the main source of variation (between fresh samples and the twice frozen–thawed ones) to the alteration of the lipidic structure. Classification models were instead built by Qin and collaborators in order to establish the freshness of red snapper fillets that, for this purpose, were subjected to two sequential freezing–thawing cycles [101]. No sample pre-treatment was required and line-scan hyperspectral images were collected signals in four different modes: visible and near-infrared (VNIR) region, short-wave infrared (SWIR) region, fluorescence mode, and Raman mode at 785 nm laser light source. Raman spectra, covering the wavenumber range of 103–2831 cm^−1^, were pre-processed to remove fluorescence background by adaptive iteratively reweighted penalized least squares; then, machine learning classifiers belonging to six categories (i.e., decision trees, discriminant analysis, Naive Bayes classifiers, support vector machines, k-nearest neighbor classifiers, and ensemble classifiers) were used and compared for all the spectral data in three different datasets (full spectra, first ten components of PCA, and bands selected by sequential feature selection method). The overall accuracy of the models was compared and calculated by averaging the errors over the two-fold cross-validation procedure. The obtained results demonstrated a better ability in classifying fish freshness for the VNIR reflectance mode (between 98.1% and 99% of accuracy when the linear and quadratic discriminant classifiers and the quadratic and cubic SVMs were used) and SWIR reflectance mode (99.99% of overall accuracy obtained by the subspace discriminant classifier) when compared to fluorescence and Raman modes (always less than 80% accuracy). The better ability is related to the higher sensitivity of VNIR and SWIR modes to the water content changes that follow the freezing and the thawing cycles. Nevertheless, the reported study confirmed the strong influence of lipid structures on the major Raman peaks to which the progressive change in the fish tissue in the freeze–thaw process is related.

### 4.4. NMR Spectroscopy

Nuclear magnetic resonance (NMR) spectroscopy is a powerful fingerprinting technique that is capable of investigating the molecular structure of the constituents in food, exploiting the magnetic properties of specific atomic nuclei under the application of a magnetic field [102].

In the field of fish products characterization, for example, proton NMR relaxometry has proven to be a powerful technique to provide information on inherent muscle water and water-holding capacity (WHC), which are fundamental parameters in perception during food consumption [103]. In this regard, it is interesting to highlight the study of Duflot and collaborators [104] on the monitoring of the Low-Field (LF) ^1^H NMR T_2_ (proton transverse relaxation signal) relaxation rate in minced hake with different thermal treatments (fresh, frozen, and cooked) taking into account all other parameters that can affect this kind of signal (pH, NaCl, and protein concentration). The authors correlated T_21_ (not significantly correlated), T_22_ (R^2^ = 0.469, significant at *p* = 0.05) relaxation times, and their corresponding maximum amplitudes A_21_ (R^2^ = 0.774, significant at *p* = 0.01) and A_22_ (R^2^ = 0.760, significant at *p* = 0.01) with WHC, and they also found that WHC decreased and that the relaxation rate of the major component increased upon frozen storage [104]. Differently, the study of Shumilina and others is more pertinent to the discrimination between fresh and thawed products. They applied an NMR metabolomics approach to the analysis of Atlantic salmon during the two different storages, observing significant variations of fumarate and phenylalanine concentrations and the formation of aspartate after thawing [42]. In particular, for each salmon, one fillet was kept at 4 °C (fresh sample) and one fillet was frozen at −40 °C for 16 h (frozen/thawed samples). These latter samples were thawed the following day and kept at 4 °C for 18 days. On day 5, 6, 7, 9, 11, 14, and 18, NMR measurements were performed on both sample sets. PCA performed on NMR variables, selected (from 258 chemical shifts to 19) with an ANOVA test (*p* < 0.05), showed along PC1 a clear grouping tendency of fresh and frozen–thawed samples starting from the second day after thawing. Freezing/thawing caused an increase of fumarate and phenylalanine in stored salmon. Moreover, the technique was able to recognize fish sampled from different packages and to detect thawing, even if the freezing occurred at different time points after slaughter. The authors proposed the formation of aspartate as a marker of the freezing/thawed process of salmon. Further examples of application of NMR spectroscopy are reported in Table 2.

### 4.5. Impedance Spectroscopy

Impedance spectroscopy has shown high potentiality to evaluate seafood freshness, since it allows detecting, with good sensitivity, the changes in the tissue resulting from the freezing–thawing process. In addition, it could be a suitable technique for quality control at different steps of the production chain, being portable, non-destructive, and easy to use. Impedance analyses focus on the electrical properties of the samples that, in biological tissues, depend on the distribution of the extra/intracellular fluid, which has a resistive function, and of the cell membranes to which a capacitive component is attributed [105]. Therefore, it is easy to deduce that the damages and the destruction of the cell membranes, following the freezing–thawing cycles, have an impact on the impedance signals. The analysis consists of applying an alternate electric signal in a variable range of frequencies and in collecting the corresponding output at the electrode. The signal is elaborated to express impedance through the module (voltage and current intensity ratio) and the phase (a frequency-dependent quantity that explains the phase differences between voltage and current signals) versus frequency. Due to the sensitivity of the technique, it is crucial to find the most suitable experimental settings depending on the different nature of samples, e.g., temperature, the analysis depth, the orientation, and the type of probe. Methods based on impedance spectroscopy were applied to different fish species. The studies reported in Table 2 focused on establishing appropriate experimental settings for fish tissue aiming to set up a method that is able to discriminate fresh samples to those subjected to different frozen storage times and freezing–thawing cycles. Although the reported methods should be properly validated, the studies provide evidence of the importance of the type and design of the probe to achieve satisfying outcomes depending on the row material. In detail, although satisfying results were not obtained with an arrowhead electrode in the case of sea bream (*Sparus aurata*) [24], the same probe proved to be efficient in discriminating between fresh and frozen–thawed salmon samples [25]. Indeed, the differences in the structure and composition could strongly affect the electrical properties, demonstrating the need to test different sensors in the case of different fish species. In a recent article, Sun and collaborators evaluated the possibility of impedance spectroscopy to be used for a quantitative prediction of the Atlantic salmon freshness [23]. Firstly, PCA scores were used to build LDA, SVM, and back propagation artificial neural network (BPANN) models in order to discriminate fresh and frozen–thawed samples; 100% in classification ability was obtained for all the approaches in the training and prediction set (33% of the samples). Moreover, a PLS model was employed to predict the total volatile base nitrogen value, which is an important parameter related to fish freshness. The good correlation coefficients (0.9447 in calibration 0.9387 in test) of the validated model (one-third of the samples in the test set) demonstrated that impedance property could be used to predict salmon freshness easily and rapidly. This achievement surely paves the way for possible future investigations and applications.

## 5. Conclusions and Future Prospects

As substituting frozen–thawed muscle foods for fresh products is considered a fraudulent practice, the establishment of a rapid and reliable method for distinction between fresh and thawed products is essential. Our bibliographic survey of publications shows that various enzymatic, histological, and other traditional methods have been tested to differentiate fresh from frozen–thawed products with variable success rates. Most of these techniques are known to share certain common limitations, such as destructiveness and the time-consuming nature of measurements.

The reviewed studies showed that most spectroscopic techniques in conjunction with advanced methods of data analysis have a great potential for detecting fraudulent practices of substituting fresh fish or other seafood products by frozen–thawed products. Indeed, according to the available literature data published during the last 20 years, different spectroscopic techniques (e.g., Vis/NIR, fluorescence, Raman, HSI, NMR, and impedance spectroscopy) have the ability to help fight against this type of food fraud. These techniques are rapid, cost-effective, and do not destroy the sample under analysis, making them promising possible solutions to replace conventional techniques in daily routine and screening analysis. Although the scientific literature on spectroscopy is still strongly dominated by publications presenting proof-of-concepts and feasibility studies, some applications [28,30,95] involving samples moving on conveyor belts tend to convey the impression of a possible applicability of these techniques in the real industrial environment.

However, the translation of spectroscopic techniques into real applications in the food industry is still hindered by some issues. The reluctance of the food industry to adopt new technologies and the investment costs for these technologies may be among the main obstacles for a wider implementation of spectroscopic techniques as routine analytical methods. The potential of hyperspectral imaging techniques can be recognized on the basis of the articles reviewed in this paper. The development of hyperspectral imaging coupled with new software that is able to process the data in real time opens a completely new era of monitoring and control in the food industry. NIR spectroscopy, applied alone or in combination with imaging modalities (i.e., hyperspectral imaging), is the most investigated technique. Especially fluorescence imaging studies should be carried out in order to take advantage of both the high sensitivity of fluorescence and the spatial dimension provided by the imaging. Although the other spectroscopic techniques have shown promise, their overall potential has so far been largely ignored. Therefore, more research studies are expected in this area in the future. Progress in instrumentation and the marriage of spectroscopy with chemometrics has already led to high resolution and portable instruments, as well as hand-held devices, while more consumer-friendly designs, such as the development of lab-on-smartphone platforms is expected with further progress. More research on the miniaturization of analytical systems and equipment cost of emerging techniques is needed. Another possible direction could be integrating multiple techniques and combining the outputs of multiple instrumental sources to overcome specific limitations associated to each technique. Such an approach has become more feasible recently as a result of advances and developments achieved in the field of chemometry and data analysis as well as the application of data fusion; the latter has become an active research topic in recent years.

## Figures and Tables

**Table 1:** Main spectroscopic techniques and their main characteristics.

Type of Spectroscopy	Wavelength Region	Wavelength Limits	Type of Transition	Advantages/Disadvantages
Absorption, emission, and fluorescence	Ultraviolet	10–380 nm	Bonding electrons in molecules	Accuracy, sensitivity/sample preparation
Absorption, emission, and fluorescence	Visible	380–750 nm	Bonding electrons in molecules	Accuracy, sensitivity/limited range
Absorption	Near-infrared	13,000–4000 cm^−1^	Vibrational position of atoms in molecular bonds	Fast, no sample preparation/non-specific, water interferes, calibration
	Mid-infrared	4000–200 cm^−1^		Fast, specific for functional groups/water interferes
	Far-infrared	200–10 cm^−1^		Suitable for studying the anion–cation interaction/complex spectrum, difficult interpretation
Nuclear magnetic resonance	Radio wave	1–1000 m	Nuclei orientation into a magnetic field	Accuracy/sample preparation, costs

**Table 2:** Further application of spectroscopic techniques for the assessment of quality changes in fish and fishery products during freezing, frozen storage, and thawing.

Type of Food	Authenticity Issue	Analytical Technique	Modelling Method	Reference
**UV-Vis and Fluorescence Spectroscopy**				
Whiting fillets	Fresh/frozen–thawed	FFFS	PCA, FDA	[40]
Cod, Mackerel, Salmon and Whiting fillets	Monitoring of fish freshness	FFFS	PCA, Mahalanobis distance method	[41]
Horse mackerel fillet	Prediction of post-mortem changes in frozen fish	EEM	PLSR	[76]
Horse mackerel fillet	Prediction ATP content in early stages post-mortem fish	EEM	PLSR	[77]
Whiting fillets	Monitoring fish freshness under different refrigerated conditions	FFFS	PCA, FDA	[78]
Japanese dace fish	Monitoring fish freshness during storage	EEM	Linear/exponential regression	[79]
Japanese dace fisheye	Prediction standard freshness index of k-value	EEM	PLSR	[80]
Japanese dace fish	freshness	UV-Vis	SVM, LDA, SIMCA	[81]
**Infrared Spectroscopy and Hyperspectral Imaging**				
Cod	Freezing history	Vis/NIR HSI	PCA	[28]
Cod	Fresh/frozen–thawed	Vis/NIR HSI	PCA + Rosenblatts perceptron	[30]
Swordfish	Fresh/frozen–thawed	NIR/Vis-NIR	PCA + multivariate binary logistic regression	[64]
Tuna	Fresh/frozen–thawed	Vis/NIR	PLS-DA	[82]
Horse mackerel	Fresh/frozen–thawed	NIR	PCA, MLR	[83]
Red sea bream	Fresh/frozen–thawed	Vis/NIR	PCA-LDA, SIMCA	[57]
Grass carp	Fresh/frozen–thawed	Vis/NIR HSI	SIMCA, LS-SVM, PNN	[84]
Several species	Fresh/frozen–thawed	NIR	PLS-DA	[85]
Goatfish	Fresh/frozen–thawed	Vis/NIR	PLS-DA; Multi-block PLS-DA	[86]
Atlantic salmon	Fresh/frozen–thawed	Vis/NIR Vis/NIR HSI	PLSR, kNN classifier	[29]
**NMR Spectroscopy**				
Several species (fish)	Effects of freezing, thawing, storage time and interaction between temperature, time, and freezing rate	LF ^1^H NMR	Several techniques	[87]
Atlantic salmon fillets	Monitoring of metabolites during cold storage and estimation of freshness indices	High-resolution NMR	-	[88]
Hake fillets	Monitoring of consequences of different freezing and storage conditions	Low-field NMR T_2_ relaxometry	-	[89]
Hake fillets	Quality changes and estimation of freezing storage time	Low-field NMR T_2_ relaxometry	PCA, PLSR	[90]
**Impedance Spectroscopy**				
Sea bream	Fresh/frozen samples, discrimination between different storage time and number of freezing cycles	Impedance spectroscopy	PCA-Stepwise LDA	[24]
Salmon	Fresh/frozen–thawed, effect of freezing storage times and number of freezing cycles	Impedance spectroscopy	PCA-Stepwise LDA	[25]

EEM; Excitation–Emission Matrix, PLSR; Partial Least Squares Regression, FFFS; Front-Face Fluorescence Spectroscopy, PCA; Principal Component Analysis, FDA; Factorial Discriminant Analysis, ANOVA; Analysis of Variance, Vis/NIR; Visible/Near Spectroscopy, HSI; Hyperspectral Imaging, kNN; k-Nearest Neighbor Classifier, SVM; Support Vector Machine, LS-SVM; Least Square Support Vector Machine, LDA; Linear Discriminant Analysis, SIMCA; Soft Independent Modeling of Class Analogy, UV-Vis; Ultraviolet Visible, PLS-DA; Partial Least Square Discriminant Analysis, PNN; Probabilistic Neural Network, MLR; Multiple Linear Regression, NMR; Nuclear Magnetic Resonance, MRI; Magnetic Resonance Imaging.
